# Comparative Cranial Joint Variation in Three Different Lizards: Impact of Feeding Habit

**DOI:** 10.3390/life15010090

**Published:** 2025-01-13

**Authors:** Elif Yıldırım Caynak, Kamil Candan, Yusuf Kumlutaş, Ahmet Gökay Korkmaz, Sezen Birlik, Dudu Mertgenç Yoldaş, Serkan Gül, Çetin Ilgaz

**Affiliations:** 1 Department of Biology, Faculty of Science, Dokuz Eylül University, 35220 İzmir, Türkiye; yildirim.elif@deu.edu.tr (E.Y.C.); kamil.candan@deu.edu.tr (K.C.); yusuf.kumlutas@deu.edu.tr (Y.K.); ahmetgokay.korkmaz@deu.edu.tr (A.G.K.); cetin.ilgaz@deu.edu.tr (Ç.I.); 2 Fauna Flora Applied and Researcher Centre, Dokuz Eylül University, 35220 İzmir, Türkiye; 3 Department of Biology, Science, and Literature Faculty, Uludag University, 16059 Bursa, Türkiye; sezen@uludag.edu.tr; 4 Machine Drawing Construction Program, İzmir Multidisciplinary Vocational School, Department of Mechanical and Metal Technologies, Dokuz Eylül University, 35220 İzmir, Türkiye; dudu.yoldas@deu.edu.tr; 5 Department of Biology, Faculty of Arts and Sciences, Recep Tayyip Erdogan University, 53100 Rize, Türkiye

**Keywords:** cranial bones, geometric morphometric, food item, Squamata

## Abstract

The skull structure in vertebrates is closely related to feeding mode. This study examines the relationship between the cranial joint morphology variation among different lizard species [*Eumesces schneideri* (Daudin, 1802), *Anguis colchica* (Nordmann, 1840), and *Eremias suphani* (Başoğlu & Hellmich 1968)] and their feeding habit. This study investigates the cranial anatomical correlates of distinct cranial kinesis models. Different cranial joints permitting intracranial mobility have been observed among these species using histological section and whole-mount techniques. The cranial joints are similar among species that generally exhibit cranial kinesis. The stomach contents of the species were analyzed, and *E. schneideri* has the highest prey diversity among the examined species, followed by *E. suphani* and *A. colchica* in that order. The study indicated that the prey preferences differ among three lizard species. While no plant material was detected in the stomach contents of *E. suphani* and *E. schneideri*, it was detected in *A. colchica*. The diet of the three lizards consisted of various species of small arthropods such as Arachnida, Lepidoptera, Coleoptera, Formicidae, and Gastropoda. Additionally, no significant differences were detected in SVL, head, and jaw size measurements between adult males and females of each species.

## 1. Introduction

Lizards, known for their anatomical and ecological diversity, have been used as a model to investigate the causes of morphological, anatomical, and functional diversity in the evolutionary process [1,2,3]. Dietary differences create mechanical responses that reflect changes in the feeding system. A significant relationship between feeding habits and skull morphology has been demonstrated in many vertebrate species [4,5,6]. Previous studies have shown that dietary habits strongly influence skull evolution in lizards [2,7,8]. For example, lizard species that consume hard food exhibit high biting performance [9], which is a result of having elongated heads with prominent temporal crests and large jaw adductors [9].

Cranial kinesis generally refers to the relative movements between specific skull bones [10,11]. This phenomenon has been observed in a variety of extant vertebrate taxa, including fish [12,13,14,15,16], amphibians [17,18], squamates [2,10,11,19,20,21,22,23,24,25,26,27,28,29,30,31,32,33,34,35,36,37,38,39], and birds [40,41,42,43,44]. Squamates exhibit various types of cranial kinesis depending on the mobility and orientation of their joints [11]. The anatomy and functional morphology of lizard cranial kinesis have been investigated in numerous studies [11,19,20,21,22,36,39,45]. While some studies have explored this system from a developmental perspective, providing histological data, only a few have specifically examined the relationship between the histology of kinetic joints and their functional environment [33,34]. Joints are divided into three main categories according to function and structure: (1) diarthroses (synovial joints), which are joints that allow free movement; (2) synarthroses (nonsynovial joints), which are joints that do not allow movement; and (3) amphiarthroses, which are joints that allow limited movement. Synarthroses include bone-to-bone synostoses (such as sutures), which are immobile, synchondroses (defined by the presence of an intervening cartilage segment), and syndesmoses (where the connection is made through fibrous tissue) [46]. Histological analyses at the morphological level have revealed that cranial sutures in squamate skulls exhibit greater diversity than once thought [1,45]. This variation suggests the presence of different patterns of intra-cranial mobility [31,33,34,47,48]. Functional studies demonstrating cranial kinesis are more limited in lizards [49]; previous research has identified various intra-cranial movements in this taxon [11,22,50]. The presence or absence of some or all of these intra-cranial movements varies among lizard taxa [11,32].

In this study, we provide new data on diets and cranial joints of three different lizard species [*Eumeces schneideri* (Daudin, 1802), *Anguis colchica* (Nordmann 1840), and *Eremias suphani* Başoğlu & Hellmich 1968], seeking to answer the following questions: (1) Which types of prey constitute their diets? (2) Is prey size correlated with lizard body size? (3) Does cranial joint morphology, especially those of the frontal-parietal, palatine-pterygoid, pterygoid-basisphenoid, parietal supraoccipital, and quadrate joints (quadrate-articular, quadrate-pterygoid, quadrate-otooccipital) correlate with the types of dietary items of examined species?

## 2. Materials and Methods

### 2.1. Studied Species Information

A total of 37 specimens were used in this study, including 11 *Anguis colchica* (5 ♀, 6 ♂), 15 *Eumeces schneideri* (9 ♀, 6 ♂), and 11 *Eremias suphani* (6 ♀, 5 ♂). After performing linear measurements on 37 specimens, the stomachs were removed for stomach content analysis. A total of 9 specimens, with 3 individuals from each species, were used for osteological analysis, while 12 individuals, with 4 specimens from each species, were used for histological analysis. Data on the collection locations of the examined species are also provided in Table 1. The studied specimens are part of the collection housed at the Fauna Flora Application and Research Center (FAMER) of Dokuz Eylül University and were collected during fieldwork between April and September. The samples are preserved in 80% ethanol and stored in glass jars under dark conditions.

### 2.2. Osteological and Histological Analysis

Osteological analysis was conducted on double-stained skulls using alcian blue and alizarin red S, following the method described by Wassersug [51]. After removing the skin and eyes, the skulls were fixed in 10% formalin for 24–48 h, washed in tap water for 24 h, and stained with alcian blue 8 GX for 48 h. The skulls were then dehydrated using ethanol. Then, the skulls were stained with alizarin red S for 48 h, cleaned, and stored in an increasing glycerol series. The excess dye was removed, and the skulls were finally stored in absolute glycerol.

Histological sections of skulls were prepared from four samples of each species (two juveniles and two adults). The samples were decalcified in 5% nitric acid for two months, with the nitric acid solution refreshed weekly. Following decalcification, the samples were dehydrated through an ethanol series and cleared with xylene. After being embedded in paraffin overnight, the samples were sectioned into 5 µm thick longitudinal and cross-sections using a rotary microtome. Staining was performed with hematoxylin. For each species, one skull was sectioned sagittally, while the remaining skulls were sectioned coronally, all using a rotary microtome. Double-stained skulls were examined with a Leica DFC295 stereo microscope (Leica Microsystems, Wetzlar, Germany) equipped with a digital camera. Histological sections were observed using a light microscope.

The terms ‘diarthrosis (joints that allow free movement), synarthrosis (joints that do not allow movement), and amphiarthrosis (joints that allow limited movement)’ for the histological classification of the joints and ‘linear (joints where the adjoining bone edges are tightly joined in a straight or nearly straight line), stepped (joints with a more irregular or zig-zag pattern at the edges of the adjoining bones), and condyloid (joints where the oval surface of one bone fits into the elliptical cavity of another bone)’ to define their shapes were used.

### 2.3. Head Size and Shape

For all examined specimens, we took the following measurements using digital calipers (with a precision of 0.1 mm) before fixation: Snout-vent length (SVL): the tip of snout to the median edge of the anterior lip of cloacal opening; head length (HL): the tip of snout to the posterior margin of ear opening; head width (HW): at the widest point of the head; head height (HH): the greatest depth of head; and jaw length (JL): the tip of the snout to the labial commissure. Before conducting statistical analysis, it is essential to check whether the data follow a normal distribution, using the Shapiro–Wilk test. Spearman correlation was applied to test the relationship between SVL and prey size within species. Additionally, a Kruskal–Wallis test was conducted to analyze whether there were statistically significant differences in SVL and prey size among the species. Sexual differences in body size (SVL) and head and jaw size (HL, HW, HH, and JL) were determined using Mann–Whitney U tests. All statistical analyses were performed using SPSS 22.

### 2.4. Diet Composition and Prey Characteristics

A total of 35 specimens of each species were dissected; their digestive systems were removed and stored in 70% ethanol. Stomach contents were placed in petri dishes and examined under a stereomicroscope to identify the food items ingested, generally at the family/order level. Any plant material present was classified into broad categories (such as fruit, seeds, and leaves). Prey items are also classified according to their hardness and ability to fly [52,53,54]. The length and width of each prey item were measured with the aid of graph paper, and the volume was estimated by the prolate spheroid formula:

V = 4/3π (length/2) × (width/2)^2^

We used the Shannon–Wiener index to quantify the dietary diversity of the lizards [55,56].

H = −∑[(pi) × log(pi)](1)

where: *H*’ = the value of the Shannon–Wiener diversity index, *Pi* = the proportion of the ith species, and *s* = the number of species in the community.

## 3. Results

A summary of cranial joint structure in the three different lizards examined is given in Table 2.

### 3.1. Cranial Joint of Species

#### 3.1.1. Frontal-Parietal Joint

**Figure 1:**
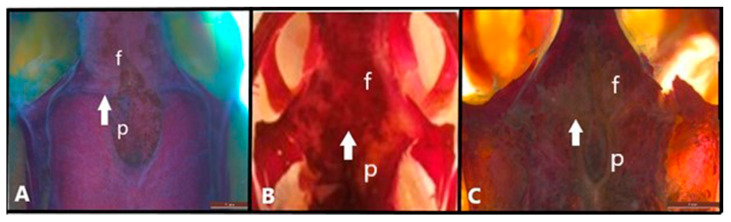
Frontal-parietal joint-mesokinesis (dorsal view) in (**A**). *Anguis colchica*, (**B**). *Eumeces schneideri* and (**C**). *Eremias suphani*. The arrow shows the frontal-parietal joint. f, frontal; p, parietal. Scale is 1 mm.

The mesokinetic joint in the frontal-parietal articulation examined histologically, showed similarities as a syndesmosis among the three species. In terms of joint shape, it was observed to be linear in *Anguis colchica*, while the other two species were observed to have a stepped configuration (Figure 1). Moreover, the broadest and most robust postfrontal bone is observed in *Eremias suphani* and *Eumeces schnederi*. In *A. colchica*, it is narrower and exhibits a triangular morphology. The postorbital bone, in contrast, is slender and extends toward the squamosal in *Eumeces schneideri* and *A. colchica*, while in *E. suphani*, it has a more triangular shape.

#### 3.1.2. Palatine-Pterygoid Joint

**Figure 2:**
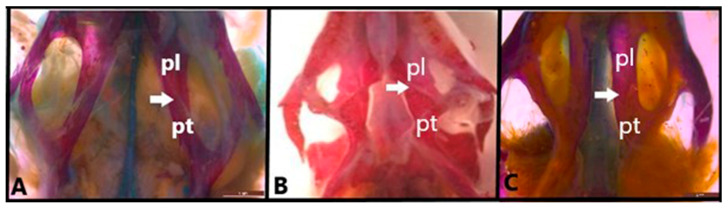
Palatine-pterygoid joint-hypokinesis (ventral view) in (**A**). *Anguis colchica*, (**B**). *Eumeces schneideri* and (**C**). *Eremias suphani*. The arrow shows the palatine-pterygoid joint. pl, palatine; pt, pterygoid. Scale is 2 mm.

In *Anguis colchica*, *Eumeces schneideri*, and *Eremias suphani*, the pterygoid and palatine bones are connected through interdigitation and syndesmosis, contributing to the overall hypokinetic structure of the cranium (Figure 2).

#### 3.1.3. Pterygoid-Basisphenoid and Parietal-Supraoccipital Joints

**Figure 3:**
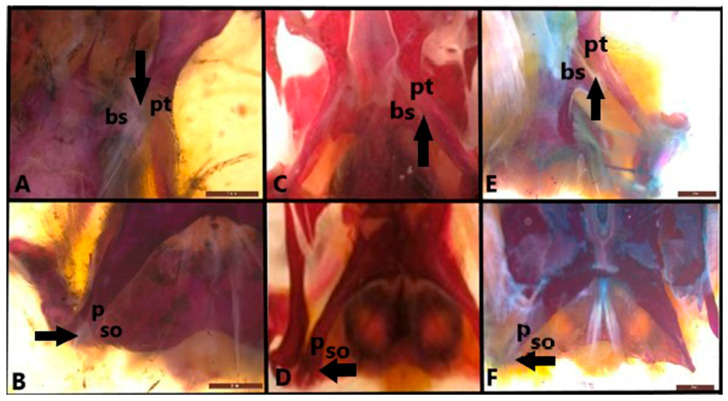
Pterygoid-basisphenoid and parietal-supraoccipital joints-metakinesis (ventral view) in (**A**,**B**). *Anguis colchica*, (**C**,**D**). *Eumeces schneideri* and (**E**,**F**). *Eremias suphani*. The arrow shows the pterygoid-basisphenoid and parietal-supraoccipital joints. bs, basisphenoid; p, parietal; pt, pterygoid; so, supraoccipital. Scale is 2 mm.

Two joints are developed in the metakinetic skull: pterygoid-basisphenoid and parietal supraoccipital (Figure 3). In the three examined lizard species, the pterygoid and basisphenoid connection is defined as a ventral metakinetic articulation, allowing movement between the neurocranium and the palate. The articulation in question occurs in the synovial joint. The connection between the parietal and supraoccipital bones is defined as the dorsal metakinetic joint. The two bones are connected to each other by a syndesmosis.

#### 3.1.4. Quadrate Joint

**Figure 4:**
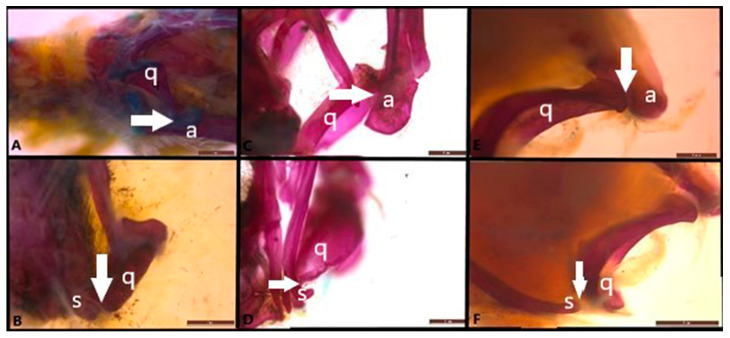
Quadrate joint-streptostyly (ventral view) in (**A**,**B**). *Anguis colchica*, (**C**,**D**). *Eumeces schneideri* and (**E**,**F**). *Eremias suphani*. The arrow shows the quadrate joints. a, articular; q, quadrate; s, squamosal. Scale is 1 mm.

The quadrate bone is located at the posterolateral end of the skull, providing a connection between the braincase and the mandible and articulating with the articular bone of the mandible (Figure 4). The quadrate connection contains three different movable joints. In all three species, the posterodorsal articular surface of the quadrate bone articulates with the otooccipital bone via a fibrous connection. Ventrally, it forms a synovial connection with the articular bone. The quadrate bone is articulated ventromedially with the quadrate process of the pterygoid bone by fibrous tissue.

### 3.2. Diet of Species

A total of 67 food items in analyzed stomachs were identified and eight prey categories, mostly arthropods were recognized (Table 3). The most common items in the stomach contents of *Anguis colchica* are plant materials and the larvae of Lepidoptera. The species with the widest prey diversity was *Eumeces schneideri*, with taxa belonging to six different groups: Coleptera, Hymenoptera, Diptera, Lepidoptera, Blattodea, and Araneae. The food items of *Eremias suphani* are mostly Araneae and Hymenoptera. The dietary breadth differs among the species, with the highest diversity in *E. schneideri* (Shannon-Wiener diversity index values are 0.77 for *A. colchica*, 3.54 for *E. schneideri*, and 1.28 for *E. suphani*). It was determined that the species whose stomach contents were analyzed in the study were omnivorous or insectivorous (Table 3). The diet of *A. colchica* includes hard and fibrous plant material such as leaves and stems. For this reason, *A.colchica* is considered omnivorous; *E. schneideri* and *E. suphani* were determined to be insectivorous according to their stomach contents.

The monthly changes in the average length of the materials observed in the stomach contents of the species are given in Table 4. Since the samples examined are FAMER collection materials, our analyses were limited to the period between April and September. The three prey taxa that contribute the most to the diet during these months are Lepidoptera, Hymenoptera, and Araneae. Since monthly diet composition and prey selection did not differ between sexes within the species, data for both sexes are presented together. When looking at the diet of all three species, it is seen that prey size is maximum in June–July; April and September were seen as months with relatively smaller prey items.

There is regional variation of the prey encountered in the stomach contents of the species (Table 1). Samples of *Aanguis cochica* were collected from the Black Sea Region of Türkiye, and it was detected that Lepidoptera and plant materials predominate in the diet of *A. colchica*. Specimens belonging to *Eumeces scheideri* were collected from the Aegean and Southeastern Anatolia Regions of Türkiye and were determined to have a diet containing mostly Hymenoptera, Lepidoptera, and Araneae members. Samples of *Eremias suphani* species were collected from the Eastern Anatolia Region of Türkiye, and a diet consisting mostly of Hymenoptera and Araneae members was observed.

**Figure 5:**
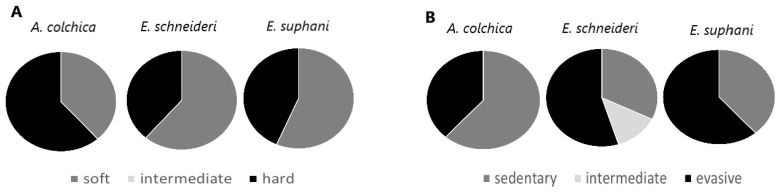
Proportions of the prey items of three examined species in relation to their hardness (**A**) and evasiveness (**B**).

In terms of prey hardness (Figure 5), among the three species examined, *Anguis colchica* predominantly consumed harder prey (62%). In contrast, *Eremias suphani* and *Eumeces schneideri* primarily fed on softer prey, with 57% and 59%, respectively. In terms of prey evasiveness (Figure 5), *A. colchica* primarily fed on sedentary prey (62%), whereas *E. suphani* and *E. schneideri* predominantly consumed evasive prey, with 57% and 55%, respectively.

### 3.3. Statistical Differences Between Species

The mean SVL, the head and jaw size measurements of adult males did not differ significantly from that of adult females in the three examined species (Mann-Whitney U test, *p* > 0.05) (Table 5). Moreover, a positive correlation between SVL (snout-vent length) and prey size was observed only in *Anguis colchica* (Spearman correlation, *p* < 0.01). However, no correlation was detected between SVL and prey size in *Eumeces schneideri* and *Eremias suphani* (Spearman correlation, *p* > 0.05). There was a statistically significant difference in SVL and prey size among the species (Kruskal-Wallis test, *p* < 0.001).

## 4. Discussion

The species examined here include both limbed and limbless species, entailing significant differences in body shape and size. Morphologically, there is a remarkable evolutionary process between limbed and limbless forms, and the modifications in this process are hidden in the skeletons of forms with snake-like body structures [57]. In addition to all of this, the fact that this group lives in a wide variety of habitats also affects their body plans. In the current study, *Anguis colchica* represents a lizard species with a snake-like morphology, *Eumeces schneideri,* a short-limbed, long-bodied form, and *Eremias suphani,* one with well-developed legs.

Of the seven cranial joints examined, five are syndesmosis, and two are synovial joints. Two of the synovial joints (quadrate-articular and pterygoid-basisphenoid) and the syndesmotic frontal-parietal joint are commonly present among lizards (Table 2). Other joints are histologically similar among the species examined here. The kinesis type of *Anguis colchica* is described as streptostyly and has two different types of joints that create quadrate movement: the synovial connection to the articular bone and the syndesmosial connection to the otoccipital and pterygoid bones. Streptostyly articulation varies among taxa [23,31]. These differences are related to feeding behavior. The metakinetic joints are similarly structured across all the examined species, with a syndesmosis joint between the parietal and supraoccipital bones, allowing for some degree of movement between these two bones. Mesokinetic movement is facilitated by the syndesmosis articulation between the frontal and parietal bones in the examined species. However, it should be noted that in *Eremias suphani* and *Eumeces schneideri*, the movement at the mesokinetic joint may be somewhat constrained due to the postfrontal bone, which is broader and approximately quadrangular in shape, located on the roof of the skull.

The diet of the examined species generally consists of Arthopoda. Van Damme [58] classified species as omnivorous if more than 5% of their diet consisted of plant material. Approximately 53.8% of the gut content was plant material in *Anguis colchica*. Due to the fossorial nature of species belonging to the genus of *Anguis*, it is very difficult to observe their diet choices in the field. For this reason, the number of studies on the diet of species in this group is insufficient. Pedersen et al. [59] showed that *A. fragilis* preyed upon worms, slugs, snails, millipedes, bees, ants, and insect larvae. Luiselli [60] noticed that *A. fragilis* generally feeds mostly on worms and snails. The current study revealed that *A. colchica* is different from *A. fragilis* in terms of diet and that it also feeds on plant materials in addition to insects. Luiselli [60] reported that *A. fragilis* primarily feeds on nocturnal and rain-active prey, such as earthworms and snails. However, in the case of *A. colchica*, only a single gastropod was detected in the stomach contents. Despite the fact that the collection sites of *A. colchica* are located in the wetter and more humid regions of Türkiye, this species did not appear to consume these prey items preferentially. Fretey [61] documented that *A. fragilis* preys on vertebrates, including lizards and snakes. In contrast, the analysis of 11 *A. colchica* stomachs revealed only three distinct prey categories, with no vertebrate remains detected. Huang et al. [62] investigated the diet structure and effect of *Eremias argus* on grasshoppers. According to this study, the food content of *E. argus* species includes grasshoppers, bees, ants, spiders, and insects. Therefore, the diet structure of *E. suphani* and *E. argus* appears to be comparable. The diet content of *Eumeces chinensis* was detected to consist of Orthoptera, Coleoptera, Lepidoptera, Diptera, Hymenoptera, Isoptera, Blattodea, Hemiptera, and Plecoptera [63]. The diet content is similar to that of *E. schneideri*. Hamilton and Pollack [64] investigated the diet of Georgian species. Among these species, *Eumeces inexpectatus* (currently *Plestiodon inexpectatus*) exhibited the highest frequency of beetles and orthopterans in its stomach contents. The average body length of the examined individuals was reported as 63.3 mm, and the remains of a lizard species, along with shed skin, were detected in two of the stomachs. The prey categories with the highest frequency of occurrence in the stomach contents, in order, were Orthoptera = Coleoptera > spiders > undetermined insects > lizard cast skin > Lepidoptera = Lizard > Centipede > Hemiptera > Mollusca. In contrast, eleven stomachs of *Eumeces laticeps* (currently *Plestiodon laticeps*) were examined, with an average body length of 92 mm. Unlike *E. inexpectatus*, four individuals contained lizard remains in their stomachs. Additionally, orthopterans and spiders were observed in three stomachs, a caterpillar and a snail in one stomach, and insect fragments were present in the remaining stomachs. The diet of the species examined in this study generally includes taxa belonging to the Insecta; no members of the Crustacea were detected.

In the current study, it was observed that the diets of *Eumeces schneideri* and *Eremias suphani* were generally similar. The dietary needs of both species are generally met by insects. However, the diet of *Anguis colchica* differs. The presence of plant material in the diet of *A. colchica* suggests that this species has a broader dietary range and enhanced ability to find food in various habitats. The inclusion of plant matter in its diet may represent an important adaptation that aids survival, as regions with abundant vegetation and moisture provide more food resources. Moreover, the Western and Eastern Black Sea regions, where the species is found, host a wide variety of vegetation. This higher plant abundance may also have played a role in more frequent consumption of plant material. *Anguis colchica*, which has plant material in its stomach contents, has a larger body size (SVL) than *E. schneideri* and *E. suphani* (Table 5). There are differences in prey preferences based on body length (SVL) among examined species. Among the examined species, a positive correlation between SVL and prey size was observed only in *A. colchica* (Spearman correlation, *p* < 0.01), whereas no correlation was detected between these variables in *E. suphani* and *E. schneideri* (Spearman correlation, *p* > 0.05). However, a significant difference in SVL and prey size was observed among the three species (Kruskal-Wallis, *p* < 0.001). Sales et al. [65] investigated the relationship between feeding habits and predator-prey size in *Cnemidophorus ocellifer* (currently *Ameivula ocellifera*, Teiidae) and reported a positive correlation between body size and prey size, as observed in most studies of *Cnemidophorus* [66].

Feeding on hard prey is generally closely related to bite force. Previous studies showed that animals with higher bite force tend to eat larger and harder prey [67,68]. It is difficult to make a direct comparison in the literature on kinesis studies because different researchers use different techniques, and the food preferences of the samples are different. Moreover, age can affect the degree of kinesis in many species. However, many taxa within the Squamata have at least one kinesis type. The skull structure of the three species examined here shows three of the kinesis types (Mesokinesis, Metakinesis, and Streptostyly) defined by Versluys [69,70].

## Figures and Tables

**Table 1:** Data on examined species in this study.

Species	Collection Site (City of Türkiye)	Samples for Diet (N)	Samples for Morphometrics (N)	Samples for Histology (N)	Samples for Osteology (N)
*Eumeces schneideri*	Aydın Adıyaman İzmir	15	15	4	3
*Erenmias suphani*	Van	11	11	4	3
*Anguis colchica*	Zonguldak Kastamonu Trabzon	11	11	4	3

**Table 2:** Descriptions of the cranial joints in three lizard species studied in the current work.

	*Anguis cochica*		*Eremias suphani*		*Eumeces schneideri*	
Cranial Joint	Histology	Joint Shape	Histology	Joint Shape	Histology	Joint Shape
Frontal-parietal	Syndesmosis	Linear	Syndesmosis	Stepped	Syndesmosis	Stepped
Palatine-pterygoid	Syndesmosis	Stepped	Syndesmosis	Stepped	Syndesmosis	Stepped
Pterygoid-basisphenoid	Synovial	Linear	Synovial	Linear	Synovial	Linear
Parietal-supraoccipital	Syndesmosis	Linear	Syndesmosis	Linear	Syndesmosis	Linear
Quadrate-articular	Synovial	Condyloid	Synovial	Condyloid	Synovial	Condyloid
Quadrate-pterygoid	Syndesmosis	Linear	Syndesmosis	Linear	Syndesmosis	Linear
Quadrate-otooccipital	Syndesmosis	Linear	Syndesmosis	Linear	Syndesmosis	Linear

**Table 3:** Diet composition of three examined species in the current study. F = frequency of occurrence, N = number, V = volume (mm^3^).

Prey Category	Species								
	*Eumeces schneideri*			*Eremias suphani*			*Anguis colchica*		
	F (%)	N (%)	V (%)	F (%)	N (%)	V (%)	F (%)	N (%)	V (%)
Coleoptera	4 (12.9)	2 (11.1)	2627.1 (0.1)						
Diptera	5 (16.1)	3 (16.7)	11,349.2 (0.5)						
Lepidoptera	4 (12.9)	1 (5.6)	79,833.6 (37)	3 (13.0)	2 (14.3)	12,108.6 (28.7)	5 (38.5)	3 (33.3)	914.6 (6.3)
Blattodea	3 (9.7)	1 (5.6)	5361.5 (2.5)	4 (17.4)	2 (14.3)	2830.8 (6.7)			
Araneae	10 (32.3)	7 (38.9)	6863.8 (3.2)	10 (43.5)	6 (42.9)	7412.2 (17.6)			
Hymenoptera	5 (16.1)	4 (22.2)	10,9809 (50.9)	6 (26.1)	4 (28.6)	19,870.2 (47.1)			
Gastropoda							1 (7.7)	1 (11.1)	6300 (43.7)
Plant item							7 (53.8)	5 (55.6)	7213.5 (50)

**Table 4:** Monthly change in the average length of prey encountered in the stomach contents of the species (mm). (N: number of samples).

Month	*Anguis colchica*		*Eumeces schneideri*		*Eremias suphani*	
	N	Average Prey Length	N	Average Prey Length	N	Average Prey Length
April	2	13	4	21		
May			3	27		
June			6	46	6	19
July	3	19			5	21
September	6	1				

**Table 5:** Summary of morphometric characters (mm) of adult males and females of three examined species.

Linear Morphometrics	*Eumeces schneideri*		
	Adult Male	Adult Female	*p*
SVL	132.89 ± 7.63	122.02 ± 6.21	0.224
HL	28.87 ± 1.91	26.24 ± 2.36	0.529
HW	18.18 ± 2.09	16.12 ± 2.23	0.388
HH	14.49 ± 1.17	13.37 ± 0.93	0.388
JL	21.05 ± 3.43	19.41 ± 4.14	0.272
	*Eremias suphani*		
	Adult male	Adult female	*p*
SVL	64.62 ± 2.35	60.44 ± 4.11	0.126
HL	19.00 ± 1.13	18.00 ± 1.73	0.082
HW	11.10 ± 0.63	10.38 ± 1.01	0.662
HH	8.73 ± 0.77	8.33 ± 0.38	0.247
JL	13.41 ± 0.26	13.06 ± 0.06	0.082
	*Anguis colchica*		
	Adult male	Adult female	*p*
SVL	186.05 ± 9.51	129.08 ± 54.55	0.082
HL	16.88 ± 2.25	14.16 ± 4.15	0.662
HW	11.13 ± 1.62	8.20 ± 2.33	0.429
HH	9.89 ± 0.62	7.25 ± 2.14	0.247
JL	13.22 ± 1.23	8.34 ± 3.78	0.082

## Data Availability

The original contributions presented in this study are included in the article.
